# Correction to “Ubiquitin ligase UBR3 regulates cellular levels of the essential DNA repair protein APE1 and is required for genome stability”

**DOI:** 10.1093/nar/gkag012

**Published:** 2026-01-14

This is a correction to: Cornelia Meisenberg, Phillip S. Tait, Irina I. Dianova, Katherine Wright, Mariola J. Edelmann, Nicola Ternette, Takafumi Tasaki, Benedikt M. Kessler, Jason L. Parsons, Yong Tae Kwon, Grigory L. Dianov, Ubiquitin ligase UBR3 regulates cellular levels of the essential DNA repair protein APE1 and is required for genome stability, *Nucleic Acids Research*, Volume 40, Issue 2, 1 January 2012, Pages 701–711, https://doi.org/10.1093/nar/gkr744

In September 2025, the Editors were alerted to potential signs of splicing in Figures 2B, 3D, and 5B. The authors acknowledge that some redundant lanes were removed from the blots in Figures 2B and 3D. Specifically, in Figure 2B, lane 3 was deleted and lanes 2 and 4 of the original image have been spliced together. In Figure 3D, lane 2 was removed and lane 1 was spliced together with the remaining lanes 3-7.

The original data of Figures 2B and 3D are provided as supplementary files.

Revised Figures 2B and 3D with black lines disclosing the splicing are provided below.

For Figure 5B, the authors confirm that there is no splicing.

This correction does not affect the results, discussion and conclusions presented in the article. The figures have been corrected only in this correction notice to preserve the published version of record.


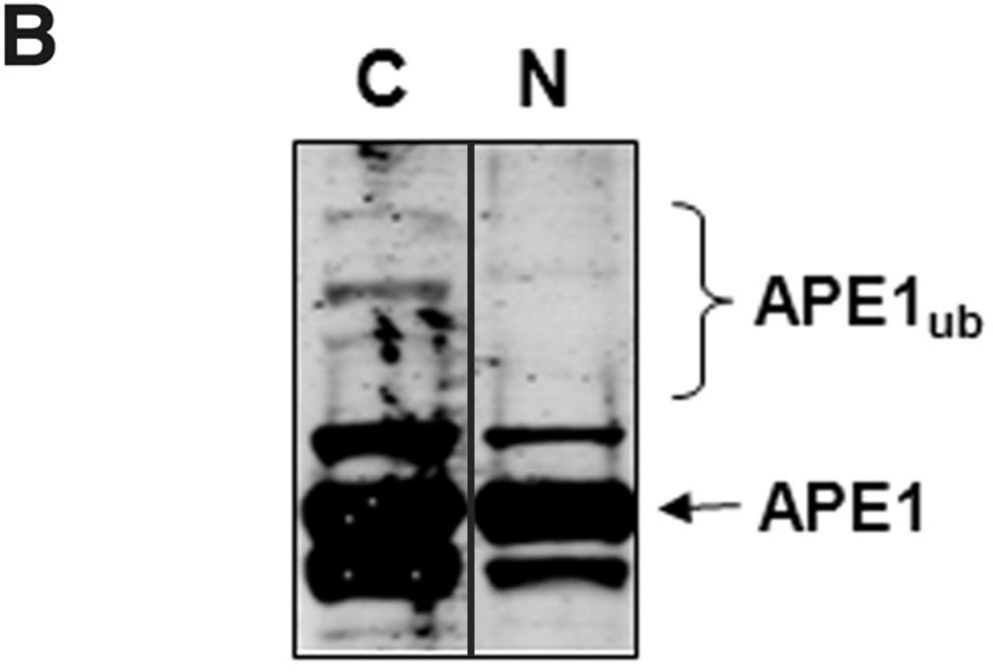


**New Figure 2B**


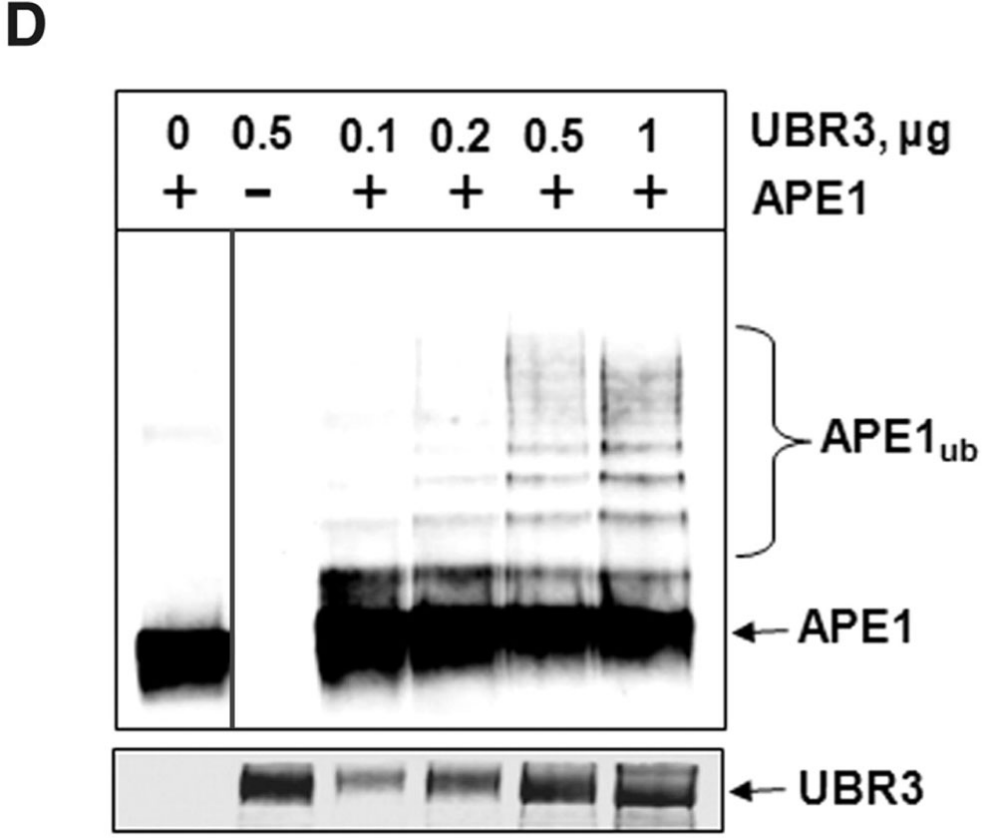


**New Figure 3D**

## Supplementary Material

gkag012_Supplemental_File

