# Supplementary material for: Correction to “Ubiquitin ligase UBR3 regulates cellular levels of the essential DNA repair protein APE1 and is required for genome stability”
Source: Nucleic Acids Res. 2026 Jan 14;54(2):gkag012. doi: 10.1093/nar/gkag012 (PMC12802881; doi:10.1093/nar/gkag012)
Supplement: gkag012_Supplemental_File [file gkag012_supplemental_file.pptx]

## Slide 1
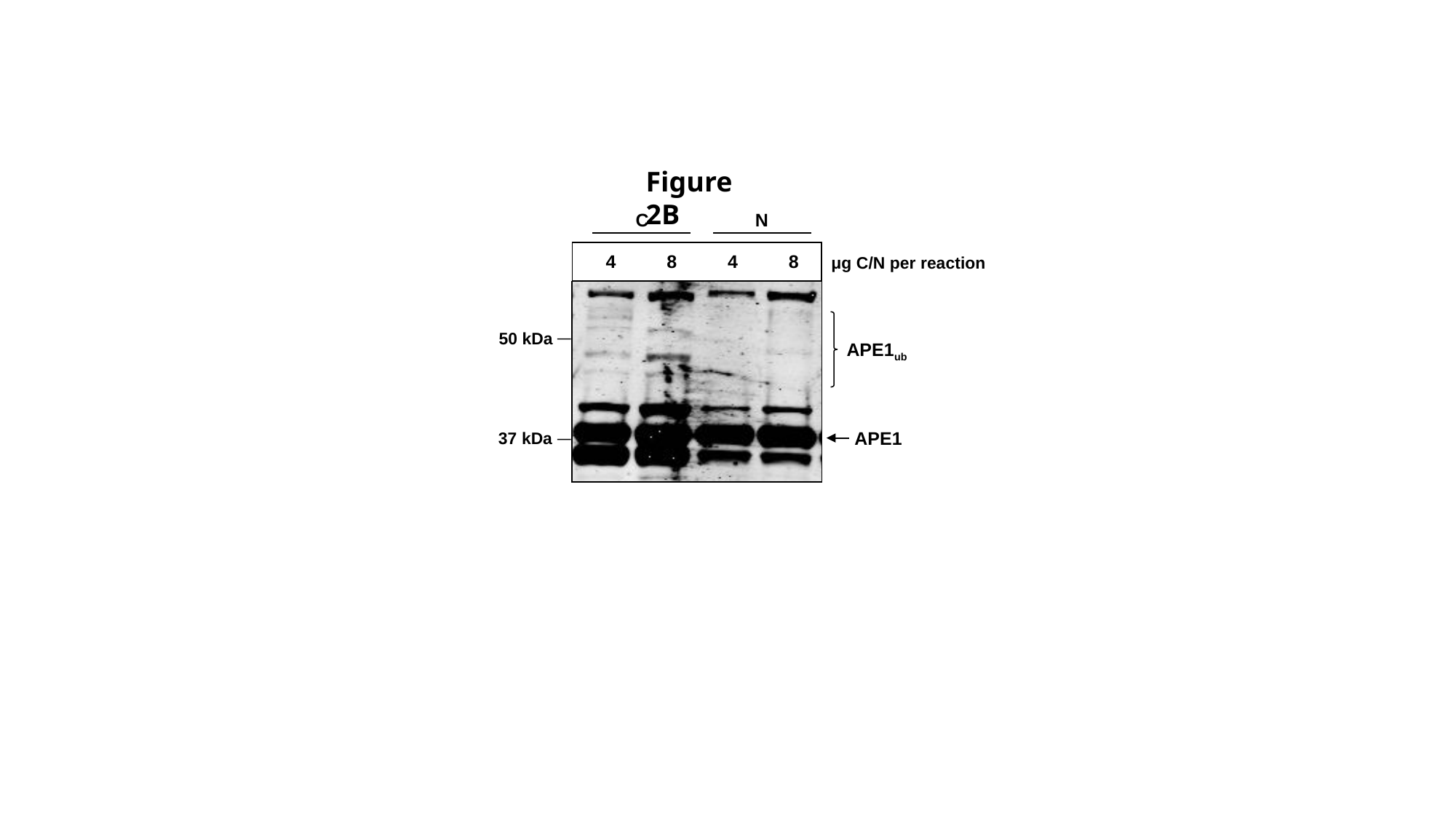

Figure 2B
C N
4 8 4 8
μg C/N per reaction
50 kDa
APE1ub
APE1
37 kDa

## Slide 2
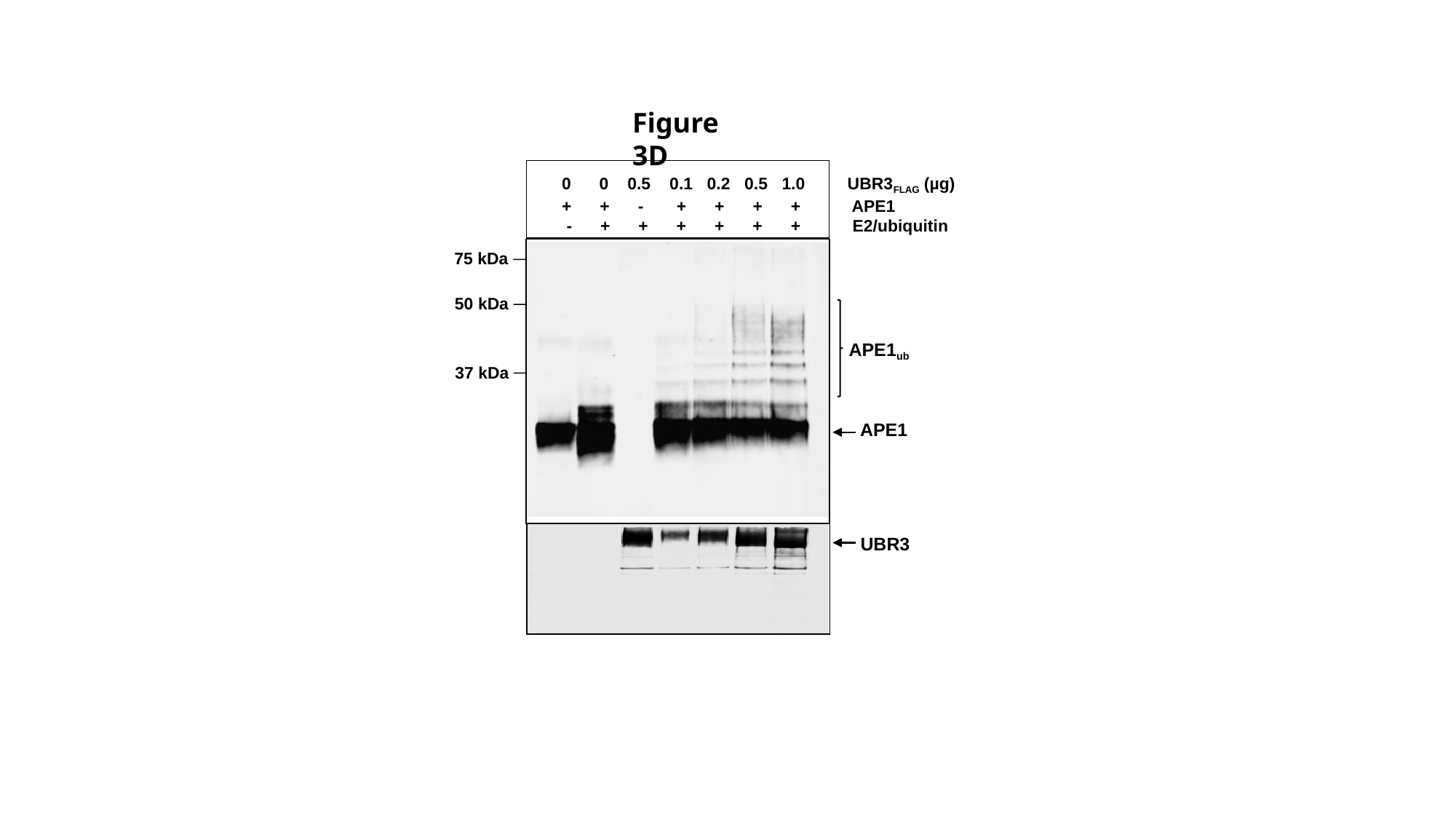

Figure 3D
0 0 0.5 0.1 0.2 0.5 1.0 UBR3FLAG (µg)
+ + - + + + + APE1
 - + + + + + + E2/ubiquitin
75 kDa
50 kDa
APE1ub
37 kDa
APE1
UBR3

## Slide 3
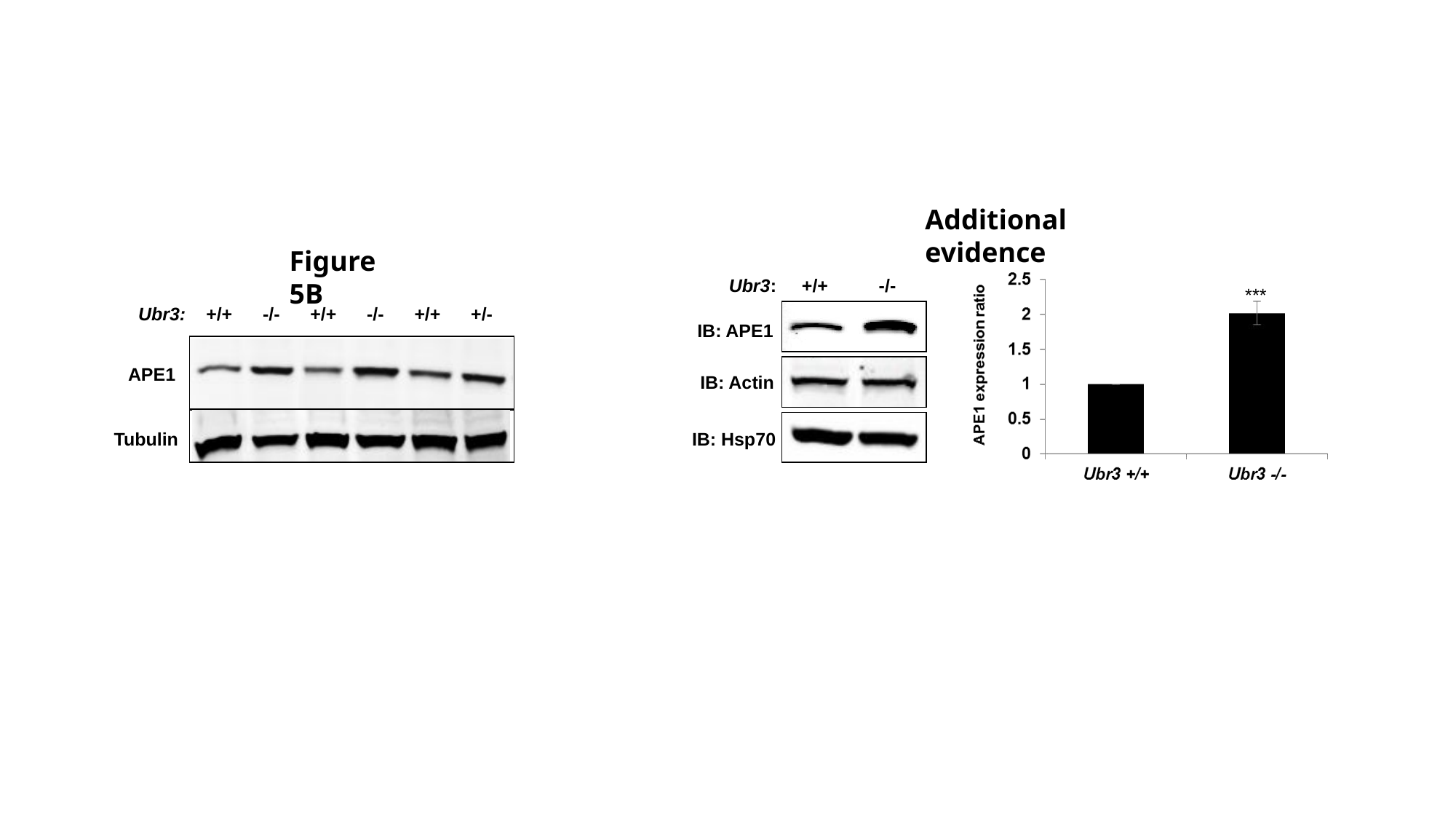

Additional evidence
***
Figure 5B
Ubr3: +/+ -/-
IB: APE1
IB: Actin
IB: Hsp70
Ubr3: +/+ -/- +/+ -/- +/+ +/-
APE1
Tubulin
